# Adaptation of the renal clinic extension-mentorship model for chronic kidney disease prevention and control into the primary healthcare system in Nigeria

**DOI:** 10.4102/jphia.v17i1.1711

**Published:** 2026-04-09

**Authors:** Akinwumi A. Akinbodewa, Olorunfemi A. Ogundele, Olumuyiwa E. Ariyo, Victoria O. Oladoyin, Korede Oluwatuyi, Adeayo O. Omotehinse

**Affiliations:** 1 Department of Internal Medicine, Faculty of Clinical Sciences, University of Medical Sciences Teaching Hospital, Ondo, Nigeria; 2 Department of Community Medicine, Faculty of Clinical Sciences, University of Medical Sciences Teaching Hospital, Ondo, Nigeria; 3 Department of Internal Medicine, Faculty of Clinical Sciences, Federal Teaching Hospital, Ido-Ekiti, Nigeria; 4 Department of Paediatrics, Faculty of Clinical Sciences, Federal Medical Centre, Owo, Nigeria; 5 Department of Paediatrics, Faculty of Clinical Sciences, University of Medical Sciences, Ondo, Nigeria; 6 Department of Public Health, Faculty of Clinical Sciences, University of Medical Sciences, Ondo, Nigeria

**Keywords:** chronic kidney disease, non-communicable diseases, integration, primary health care, nephrology clinic, universal health coverage

## Abstract

Chronic kidney disease has become a global pandemic, with its burden felt mostly in developing countries where there is a deficiency or outright lack of policy and funding for chronic kidney disease prevention and treatment. In Nigeria, its prevalence stands at about 13%, with the majority living in rural settings, far flung from nephrologists. The recent success achieved in integrating chronic kidney disease into primary healthcare has yet to receive significant funding from many governments, including Nigeria. Sustaining efforts towards disease prevention remains unachievable, with renal teams only embarking on one-off community outreaches that often fail to diagnose the disease.

**Contribution:**

In this article, the authors propose an adaptation of the *nephrology clinic extension-mentorship model*, which is designed to enable community-based kidney care that is anchored on nephrology teams acting as the fulcrum and basic functional unit. The model focuses on setting up semi-autonomous, *quasi*-renal clinics in local government areas in communities across Nigeria that will serve as platforms for sustained community engagement and participation in kidney care. The model also systematically entrenches continual screening and diagnosis of chronic kidney disease amongst at-risk groups within and outside the health centre premises through the provision of laboratory equipment and personnel that are specific for the purpose.

## Introduction

Nigeria is a member of the United Nations that adopted the primary healthcare (PHC) system, which focused on people-centred health promotion, disease prevention and treatment at the 1978 Alma-Ata declaration. This was later modified to include integration of chronic diseases and their funding into primary health centres as well as promoting community engagement at the declaration of Astana in 2018.^1,2^ Primary healthcare has improved our understanding of taking healthcare beyond the limits of healthcare workers within four walls of hospitals as in-hospital medical interventions alone have proven inadequate for achieving equitable universal health coverage for communities, especially the underserved.

Chronic kidney disease (CKD) has become a pandemic with an estimated 700–840 million people affected globally.^3^ In Nigeria, the prevalence of CKD lies between 10% and 13.9%, being driven mostly by diseases such as hypertension, diabetes and chronic glomerulonephritis.^4^ In Africa, the burden of CKD varies from nation to nation, but the risk factors and settings are similar.^5^ To date, efforts to reduce the prevalence of CKD have largely proven ineffective, especially in developing countries. Key reasons behind this include late presentation to the hospital, high costs of care, poor knowledge of CKD amongst non-nephrologists, late recognition and treatment of hypertension and diabetes mellitus, poor lifestyle, exposure to nephrotoxins as well as poor knowledge and attitude to CKD amongst patients. Of utmost importance is the limited availability of nephrologists in many African countries, with less than 300 serving a population of over 200 million in Nigeria, for instance.^6,7,8^

A major challenge facing CKD control is that it is yet to be fully incorporated into the PHC system as a standardised stand-alone component in Nigeria; hence, it does not attract government funding, unlike tuberculosis, cancer, etc. Due to limited resources, available testing materials like urinalysis strips are usually reserved for other clients like pregnant women, diabetics and children.

Indeed, recent efforts by a renal consortium on inclusion of kidney disease in the current World Health Organization (WHO) statement on major noncommunicable diseases drivers of premature mortality are beginning to yield results.^9,10^ Therefore, successful integration of CKD care into the PHC system in Nigeria can offer the opportunity to navigate mounting challenges and improve access to kidney care in the community, especially in the more populated, underserved rural areas.^11^

## Proposed adaptation of the nephrology clinic extension-mentorship model in Nigeria

In this article, we propose an adaptation of a community nephrology practice model that may form a framework for the sustainable integration of CKD prevention into the PHC system in underserved communities. In this adaptation, we aim to weave primary and secondary or tertiary healthcare services into one seamless practice with effective utilisation of the nephrology team as key players in delivering kidney healthcare outside the four walls of the hospital. By this, the following can be achieved with minimal costs: early detection of CKD and referral, attitudinal change to kidney health in the community and capacity building amongst the health centre staff.

Importantly, it will serve as a solution to the ineffectiveness of previous CKD-focused community outreaches to significantly reduce the prevalence of CKD and its attendant rise in morbidity and mortality.^9,12^ This is more likely because these outreaches were largely periodic, irregular, isolated, infrequent and short-term with long periods of dormant intervals, hence the little impact. More poignantly, these activities remain largely outside the PHC system and so were unable to receive the needed support for broad-based and sustainable impact.^13^ For instance, amongst approximately 1 million patients across 33 US health systems, only about 6.7% of those with abnormal dipstick results were recommended for confirmatory evaluation, thus leaving many with possible early CKD undiagnosed and failing to change clinical outcomes significantly, if any at all.^14^ An adaptation of the model in Nigeria will engender sustained presence of kidney healthcare personnel in communities, which can provide opportunities for lasting and significant reduction in the prevalence of CKD.

## Description of the nephrology clinic extension-mentorship model

**FIGURE 1:**
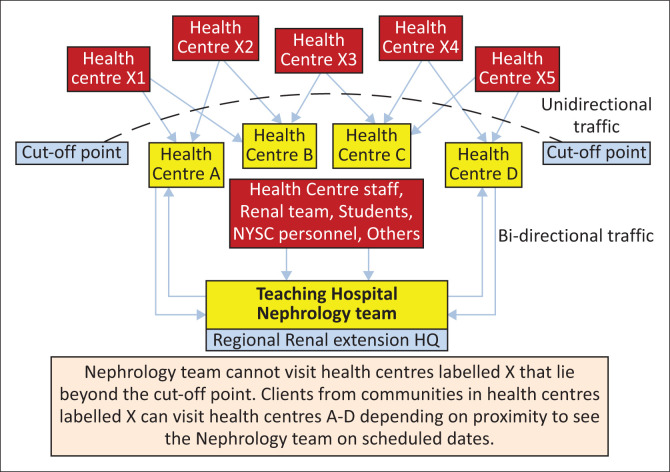
Nephrology clinic extension-mentorship model.

In the *nephrology clinic extension-mentorship model*, existing secondary and/or tertiary hospitals with the presence of at least one Nephrologist will be enlisted as a sectional renal mentoring headquarters. To each enlisted hospital will be assigned a group of health centres which fall within a specified distance from the hospital (Figure 1). This measured distance is to ensure easy accessibility of the team to only the designated centres for clinic consultation and mentoring of health centre staff and the community, reduce travel time and avoid fatigue of the personnel. These health centres will be assigned the tag, ‘Alpha units’ (labelled health centre A–D in Figure 1). The renal team will be required to visit these ‘Alpha units’ on a scheduled itinerary, ensuring that their time and presence are evenly spread amongst the centres within a specified time period.

The Alpha units are further subdivided into two: the ‘Active’ and ‘Rest’ units. An ‘Active Alpha unit’ is defined as a health centre that is currently hosting the renal team during a particular visit, whilst the adjacent Alpha units awaiting visits are the ‘Rest Alpha units’. As shown in Figure 1, people from ‘Rest Alpha units’ can visit an ‘Active Alpha unit’, thus maximising every presence of the renal team.

The second group of health centres are tagged ‘Beta units’ (labelled health centre X1–X5 in Figure 1). They are the health centres that fall outside the specified distance of coverage by the renal team from the sectional renal mentoring headquarters. It is expected that this set of health centres is served by another renal team in whose jurisdiction they fall. However, if this is not the case, the itinerary of the renal team will be made available to them via the medical officer of health (or health centre coordinators) at the various ‘Beta units’. This way, they can access renal health care in any of the ‘Alpha unit’ any time the team is available (unidirectional). In effect, provided there is sufficient spread of personnel across institutions in a given region, a ‘Beta unit’ to one renal team can simultaneously be an ‘Alpha unit’ to another renal team from another sectional headquarters and vice versa.

This system thus promotes shortened and compact road trips for the renal team, energy conservation and efficient renal healthcare coverage to communities. At an ‘Active Alpha unit’, the team is required to run clinics, train the health centre staff on early detection of CKD, ensure availability of basic tools for laboratory screening for CKD, coordinate community outreaches and establish an efficient system for early treatment interventions and prompt referral in collaboration with the medical officer of health. By so doing, the basic fundamentals of kidney care would have been passed on to the health centre personnel in a hands-on, practical method over a period of time.

## Factors that may promote successful implementation of the model

A successful adaptation of the model can be achieved by adding the following key elements:

- **Provision of support personnel to community health extension workers (CHEWs):** Despite formal training, many CHEWs in Nigeria are not formally employed by the government. A large number of them end up as poorly remunerated (and by inference, poorly motivated) volunteers in health centres.^15^ A typical primary health centre in Nigeria comprises an easy-to-access building that caters to the following: registration and data collation, consultation, education and community mobilisation, minor investigations, drug provision and early referral. It is usually headed by a Community Health Officer or senior CHEW or occasionally by a midwife. Other staffs include junior CHEWs, Environmental Health Officers and trained pharmacy and laboratory technicians. Whilst doctors are not usually posted to the health centres, there is a Medical Officer of Health (who reports to the State Primary Healthcare Board) assigned to coordinate activities in all the health centres per local government area.

The shortfall of CHEWs in the healthcare system will be addressed by the model through building a sustained pool of medical and allied healthcare students through rural posting. Adjusted school curricula can ensure that students rotating through nephrology are posted to health centres. Other potential personnel include interns (doctors, nurses, medical laboratory scientists, etc.) and National Youth Service Corp (NYSC) members who are trained in healthcare. In fact, the NYSC are already utilising medical corps members in their Health Initiative for Rural Dwellers.^16^ Also, serious steps are being taken by various groups and institutions to explore and encourage the rural medical internship.^17^ For example, in Australia, final year medical students have been undertaking 8 weeks of rural internships since 2005, with a significant impact on rural health coverage.^18,19^ The students, interns and NYSC personnel will participate in house-to-house awareness campaigns, identification of persons with risk factors for CKD, counselling and mobilisation of identified persons to the secondary or tertiary clinics. To ensure uniformity of practice and care, a manual for CKD detection, first aid and referral will be provided for all health centres.

- **Community participation in provision of accommodation and transportation:** Inadequate accommodation and amenities could pose a major hindrance to rural outreach. To overcome this, host communities will be engaged to provide low-cost accommodation. Where there is no access to telephony and the internet, routers and facilities for internet communication and telephone devices will be provided by the community, with regular online supervision by senior members of the renal team. This will serve as an added incentive. Mobile applications can also be developed to help CHEWs and junior cadre staff and *ad hoc* staff to easily identify CKD in situations where the renal team is not physically available. These applications (especially those that can work offline) can be used for disease risk assessment, education and self-monitoring. In health centres where internet access is poor or remote, use of Information, Education and Communication (IEC) materials like posters, banners, leaflets, etc., will be emphasised. Zonal representatives who will work with local transportation union to organise transportation for those in need of referral will be appointed.
- **Mobilising alternative funding source:** Underfunding, lack of political will, wastefulness and lack of accountability are factors limiting employment of CHEWs and efficient operation of health centres in Nigeria.^20^ Funds can be mobilised through collaboration with host communities, individuals and non-government bodies. Renal teams and host communities must collaborate to enshrine healthcare financing into the state or national budget. The renal team will generate real-time local data on CKD and risk factors and build a community coalition through regular meetings with the traditional leaders, local political officers, youth leaders and key community influencers. Using this platform, the renal team will help the community form a lobbyist group that will formulate how the drawn-up financial plans will align with the state priorities and determine steps to take in convincing state political officers to include this in the state budget. The local government Chairman and House of Representatives member for that community are key forces at this stage as they are most likely to know when decisions on budget and release of funds will be made. Timely use of media for public awareness and support through demonstration of early wins are added strategies for fundraising. For example, the former Minister for Health and his team were able to achieve the policy for allocation of 1% of Nigeria’s annual health budget to finance PHC using similar strategies.^21^

Specifically, in the renal clinic extension-mentoring model, funds will be pooled into an autonomous, Trust Foundation account with members drawn from the renal team, hospital management and the host community, with reports on income and expenditure rendered regularly. In every local government area, a percentage of tax revenue should be set aside for CKD prevention activities and paid into the Trust Fund. The recent Supreme Court judgement, which granted autonomy to local governments in Nigeria to manage their finances and allocate resources without state government interference, could work in favour of this model.^22^

A low-hanging fruit is communal fund mobilisation. Landlord Associations, social clubs and societies, traditional and opinion leaders can assist in mobilising people in host communities to donate to the fund. Communities can directly hire ad hoc staff for health centres and donate accommodation facilities.

To avoid abuse and wastage, funds raised shall be restricted to primary prevention activities and infrastructural maintenance. Provision of medications, dialysis or kidney transplantation shall not be covered by the fund which will be used specifically for the following: advocacy and community mobilisation, provision of educational materials, support for laboratory reagents and kits, transportation of medical personnel and students, accommodation of personnel, provision for communication (telephony and internet services) and training of personnel and supervision.

In addition to the foregoing, the key role of government is to provide for the health and safety of its people. Considerable and sustained pressure must be put on the government to ensure that legislation for CKD financing is pushed through in all states of Nigeria, as the enormous amount of resources with the government is incomparable to any other.

## Conclusion

Integrating CKD care into the PHC system has become an urgent need that can be facilitated by establishing sustained, close contact between the renal team and surrounding communities. An adaptation of the *nephrology clinic extension-mentorship model* for the integration of CKD care can be a low-cost, direct and practical medium to achieve sustainable awareness of CKD and change in community attitude to early screening and detection as well as care of CKD in communities through sustained visits to the underserved rural areas, whilst utilising relatively lesser human and capital resources.

## References

[1] World Health Organization and United Nations Children’s Fund. Declaration of Alma Ata. International Conference on Primary Health Care, Alma Ata, USSR, 6–12 September 1978. Geneva: World Health Organization; 1978. WHO/EURO:1978-3938-43697-61471.

[2] World Health Organization. Declaration of Astana: Global conference on primary health care: Astana, Kazakhstan, 25 and 26 October 2018 [homepage on the Internet]. Geneva: World Health Organization; 2019 [cited 2025 Sep 6]. WHO/HIS/SDS/2018.61. Available from: https://iris.who.int/handle/10665/328123

[3] GBD. Global, regional, and national life expectancy, all-cause mortality, and cause-specific mortality for 249 causes of death, 1980–2015: A systematic analysis for the Global Burden of Disease Study 2015. Lancet. 2016;388:1459–1544. https://doi/10.1016/S0140-6736(16)31012-1 27733281 10.1016/S0140-6736(16)31012-1 PMC5388903

[4] Ulasi II, Ijoma CK. The enormity of chronic kidney disease in Nigeria: The situation in South-East Nigeria. J Trop Med. 2010;1:1–6. 10.1155/2010/501957 PMC2896838 20613945

[5] Abd ElHafeez S, Bolignano D, D’Arrigo G, Dounousi E, Tripepi G, Zoccali C. Prevalence and burden of chronic kidney disease among the general population and high risk groups in Africa: A systematic review. BMJ Open. 2018;8(1):e015069. 10.1136/bmjopen-2016-015069 PMC5780690 29326180

[6] Adejumo OA, Akinbodewa AA, Okaka EI, Alli OE, Ibukun IF. Chronic kidney disease in Nigeria: Late presentation is still the norm. Niger Med J 2016;57:185–189. 10.1155/2010/501957 27397961 PMC4924403

[7] Konde LK, Mungati M, Akugizibwe et al. The socio-economic impact of kidney disease on African families: A scoping review. Discover Public Health. 2025;22:17. 10.1186/s12982-025-00396-x

[8] Choukem SP, Nchifor PK, Halle M, et al. Knowledge of physicians on chronic kidney disease and their attitudes towards referral, in two cities of Cameroon: A cross-sectional study. BMC Res Notes. 2016;9:29. 10.1186/s13104-016-1845-5 26781039 PMC4716638

[9] Francis A, Harhay MN, Ong ACM, et al. Chronic kidney disease and the global public health agenda: An international consensus. Nat Rev Nephrol 2024;20(7):473–485. 10.1038/s41581-024-00820-6 38570631

[10] Ashrafi SA, Schramer E, Schwingel A. Community health workers and chronic kidney disease: A global perspective. Kidney Int Reports. 2024;9(4): S242. 10.1016/j.ekir.2024.02.601

[11] World Bank. Rural population (percentage of total population) Nigeria [homepage on the Internet]. World Bank Open Data. 2023 [cited 2025 Sep 16]. Available from: https://data.worldbank.org/indicator/SP.RUR.TOTL.ZS

[12] Wong G, Bernier-Jean A, Rovin B, Ronco P. Time for action: Recognizing chronic kidney disease as a major non-communicable disease driver of premature mortality. Kidney Int. 2024;105:1144–1146. 10.1016/J.KINT.2024.05.014 38579988

[13] Ameh O, Ekrikpo UE, Kengne AP. Preventing chronic kidney disease in low and middle income countries: A call for urgent action. Kidney Int Rep. 2019;5(3):255–262. 10.1016/j.ekir.2019.12.013 32154447 PMC7056849

[14] Xu Y, Shin Ji, Wallace A, et al. Shortfalls in follow-up albuminuria quantification after an abnormal result on a urine protein dipstick test. Ann Int Med. 2024;177(11):1593–1595. 10.7326/ANNALS-24-00549 39348706 PMC11824602

[15] Federal Ministry of Health, Nigeria. Second national strategic health development plan 2018–2022 [homepage on the Internet]. Abuja: Federal Ministry of health Nigeria, 2018 [cited 2025 Oct 3]; 150p. Available from: https://health.gov.ng/doc/NSHDPIIMEPlan.pdf

[16] National Youth Service Corp Health Initiative for Rural Dwellers (HIRD). Abuja: National Youth Service Corps [cited 2025 Oct 22]. Available from: https://nysc.gov.ng

[17] McGrail MR, O’Sullivan BG, Russel DJ, Rahman M. Exploring preference for, and uptake of, rural medical internships, a key issue for supporting rural training pathways. BMC Health Serv Res. 2020;20:930. 10.1186/s12913-020-05779-1 33032604 PMC7543036

[18] Gupta TS, Muray RB, McDonnel A, Murphy B, Underhill AD. Rural internships for final year students: Clinical experience, education and workforce. Rural Remote Health. 2008;8(1):827. https://doi/10.22605/RRH827 18271675

[19] McGrail MR, Fox J, Martin P. Evaluating the importance of rural internships to subsequent medical workforce distribution outcomes: An Australian cohort study. BMJ Open. 2024;14(10):e084784. 10.1136/bmjopen-2024-084784 PMC11492955 39424396

[20] Madua AC, Osborne K. Healthcare financing in Nigeria: A policy review. Int J Soc Determinants Health Serv. 2023;53(4):434–443. 10.1177/27551938231173611 37186783

[21] Schreiber L. Making good on a promise: Boosting primary health care funding in Nigeria, 2015 – 2019 [homepage on the Internet]. Princeton, NJ: Innovations for Successful Societies; 2019 [cited 2025 Oct 26]. Available from: http://successfulsocieties.princeton.edu/publications/making-good-promise-boosting-primary-health-care-funding-nigeria

[22] Cyriacus IE, Ibrahim Y, Sada MM, Ezenwajiobi CC. Nigeria’s Local government autonomy: Issues and implications for the country’s development. J Polit Discourse [serial online]. 2024 [cited 2025 Nov 2];2(3):48–60, Available from: https://www.jopd.com.ng

